# Newborn Hearing Screening Program Implantation Analysis at a University Hospital

**DOI:** 10.1016/S1808-8694(15)30784-9

**Published:** 2015-10-19

**Authors:** Wilian Maduell de Mattos, Luciana Ferreira Cardoso, Clarice Bissani, Maria Madalena C. Pinheiro, Carla Mherlyn Viveiros, Waldir Carreirão Filho

**Affiliations:** 1 MD. Universidade Federal de Santa Catarina; 2 MSc in Medical Sciences, Laboratório de Estudos da Voz e Audição - HU/UFSC; Coordinator of the Núcleo de Fonoaudiologia do - HU/UFSC; 3 PhD. Adjunct Professor - Department of Pediatrics - Universidade Federal de Santa Catarina; 4 MSc in Human Communications Disorders - Universidade Federal de São Paulo. Speech and Hearing Therapist - Laboratório de Estudos da Voz e Audição - HU-UFSC; 5 MSc in Human Communications Disorders, Speech and Hearing Therapy - Universidade Federal de São Paulo. Speech and Hearing Therapist Laboratório de Estudos da Voz e Audição - HU-UFSC; 6 MSc in Otorhinolaryngology - PUC -RJ. Adjunct Professor of Otorhinolaryngology - UFSC

**Keywords:** hearing loss, neonatal screening, hearing

## Abstract

Hearing loss is more prevalent than other disorders found at birth. Efforts have been put up towards the early identification and treatment of hearing loss by means of neonatal hearing screening programs.

**Aim:**

prospective study with the goal of characterizing the process of implementing a Neonatal Auditory Screening Program (NASP) at a University Hospital. To analyze hearing loss diagnostic investigations in newborns, and to present proposals for NASP improvement.

**Materials and Methods:**

we studied newborns (NB) submitted to Newborn Auditory Screening (NAS) by transient evoked otoacoustic emissions (TEOE), cochlear-eyelid reflex (CER) and Brainstem Evoked Auditory Potential (BEAP).

**Results:**

we tested 625 children. In the first stage, 458 NBs passed and 155 failed. 122 NBs returned to the second stage, and 8 underwent it because they were positive for HL risk factors. 12 NBs (1.9%) were referred for diagnostic investigation. Of the 5 who returned for the BAEP, we observed HL in two NBs.

**Conclusions:**

the program tested 81.7% of the candidates. The program compliance rate was of 68.2%. In the first stage, 26.7% of the NBs failed. The program is being implemented and requires constant analyzes of its difficulties, aiming at solving them in order to turn the Universal Newborn Auditory Screening into reality.

## INTRODUCTION

Today, because of the magnitude of the losses caused by hearing impairment, this topic has received broad attention from health care authorities throughout the world. For quite some time now, the need for early detection and proper treatment is advocated, since the diagnosis of this disorder, usually late, around the third year of life,^1^, ^2^, ^3^ is based only in the child's demeanor.

The early treatment of hearing loss brings known benefits, through the use of hearing aids, all the way to a cochlear implant, in order to enhance normal language development, especially when done before six months of life.^3^

In Brazil, hearing loss early detection programs have been carried out in maternities of 22 states, following international recommendations, in a total of 237 services registered at the Grupo de Apoio à Triagem Auditiva Neonatal Universal (GATANU - support group to universal neonatal hearing screening) and some cities which have legislation establishing that it is mandatory to perform hearing screening in all newborns.^2^, ^3^, ^4^

The present investigation aims at characterizing the Neonatal Auditory Screening Program in a University Hospital, analyze the diagnostic investigation of hearing loss in newborns and present proposals to enhance neonatal hearing screening.

**MATERIALS AND METHODS**

This study was a cross-sectional contemporary cohort. The subjects were all newborns (NB) submitted to hearing screening at the hospital from March 01 to August, 31 of 2005. Three NBs were taken off the study because it was not possible to see their charts.

The study project was approved by the Ethics Committee, on December 13, 2004, under protocol # 324/04. Parents or guardians had to sign a free and informed consent form.

In order to assess hearing acuity, we used the transient otoacoustic emissions hearing accuracy device, from Madsen, Accu Screen Pro T model, produced by GN Otometrics, near the hospital discharge hour of the NBs admitted to joint quarters or after clinical stabilization. The behavioral assessment was carried out by means of studying the cochleopalpebral reflex (CPR), using an agogo as an instrument, at the time of the emissions test.

In this first step, the in-hospital tests were carried out from Monday through Friday. The untested NBs and those discharged on the weekends and/or holidays were referred to outpatient evaluation between two and four weeks of life. The tests were conducted by speech and hearing therapists. The probe for otoacoustic emissions capture was coupled to the newborns' outer ears, preferably during their natural sleep and after breastfeeding. Should the test yield a hearing alteration, it was repeated on the second stage of the program, between 7 and 15 days after hospital discharge.

On the second stage, the middle ear was assessed by means of the tympanometric curve in those patients with altered screening tests. These patients would be referred to medical assessment for new tests.

When indicated, the NB would go through a third investigation stage, being then assessed by Brainstem Evoked Auditory Potential (BEAP), by an interacoustics ABR EP15 system.

**Statistical Analysis**

The data collected was organized and analyzed by the Epidata 3.0® and Epi-Info 6.0® software. For the numerical variables, we calculated the absolute values and the variability and position descriptive measures (mean and mean standard deviation). In order to check the significance, we used the X2 (α < 5%) test.

## RESULTS

**Figure 1:**
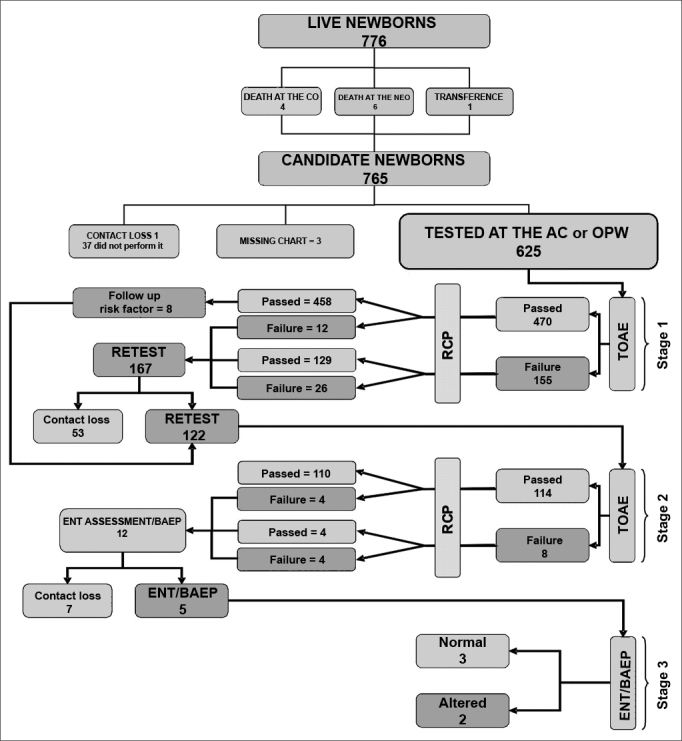
Flowchart of the program stages and the number of NBs assessed in the period.

During the study period, we had 774 live births in the maternity. We included in the study two newborns from home deliveries who were admitted after birth. One NB was transferred to another hospital. Four NBs died in the obstetric center and six died in the neonatal unit. Thus, a total of 765 NBs were eligible for neonatal auditory screening. On figure 1, we see the program stages' flowchart and the newborns evaluated during the period.

Of all the 625 patients tested, 595 (95.2%) underwent auditory testing by TOAE during their hospital stay. 30 NBs who returned to the outpatient ward were tested (4.8%). Of the 765 NBs eligible to the auditory test, 140 (18.3%) were not tested during the hospital stay and did not return to the outpatient test.

In the first Newborn Auditory Screening - NAS, the TOAE tests were normal - negative/passed - in 458 NBs (73.3%) and altered - positive/failed - in 167 (26.7%). The Cochleopalpebral reflex - CPR was altered in 38 NBs (6.1%) (Table 1).

**Table 1:** Distribution of the TOAE and CPR test results in the first stage.

CPR						
	Passed		Failed		Total	
EOAT	n	%	n	%	n	%
Passed	458	73.3	12	1.9	470	75.2
Failed	129	20.6	26	4.2	155	24.8
Total	587	93.9	38	6.1	625	100

The unilateral failure type was identified in 97 NBs (62.2%), while bilateral failure happened in 58 (37.4%) from the total of 155 emission tests that failed.

For the second NAS stage, 122 NBs participated and 8 of them who passed the first stage tests returned for having risk factors. In the retest, 114 NBs passed the TOAEs and 8 failed, and of these 5 (62.5%) failed bilaterally. The CPR was absent in 8 NBs (Table 2).

**Table 2:** Distribution of the TOAE and CPR test results in the second stage.

CPR						
	Passed		Failed		Total	
TOAE	n	%	n	%	n	%
Passed	^a110^	90.1	^b4^	3.3	114	93.4
Failed	4	3.3	4	3.3	8	6.6
Total	114	93.4	8	6.6	122	100

^a^7 of the 110 belonged to the risk factor follow up

^b^1 of the 4 belonged to the risk factor follow up.

Tympanometry was normal in 12 patients who failed the retest. These 12 patients were referred to the otorhinolaryngology department and for BAEP evaluation. The NBs born in the first 6 months of implantation, when we still did not have the test and who were waiting for it, were called in. Six NBs were located by their phone numbers recorded in their charts. One of the NBs is being followed up in another municipality. Five children came for investigation and BAEP. In these we confirmed the hearing loss in two, representing 0.32% from the total population studied (Chart 1).

**Chart 1:** Follow up of NBs who failed the 1st and 2nd stages with indication of BAEP.

NB	GA LMD/CSa	Indicador de risco	PEATE
	Risk indicator	BAEP	não realizado
1	35.7 / -	Down syndrome	Not performed
2	42.4 / -	HIV+ mother	Not performed
3	- / 33.9	Illicit drugs	Not performed
4	37.3 / -	Progeroid syndrome. craniofacial anomaly	Normal on the R and fail on the L
5	40.5 / -	Absent	Normal
6	- / 40.7	Family history	Not performed
7	38.0 / -	Family history	Normal
8	38.0 / -	Smoking	Not performed
9	34.1 / -	ICU. ototoxic. Skull anomaly. asphyxia	Bilateral retrocochlear alteration
10	37.4 / -	Down syndrome. Skull anomaly	Normal
11	- / 40.4	Syphilis. Craniofacial anomaly	Follow up in another district
12	28.6 / 28.4b	Very low weight. ICU. ototoxic	Not performed

^a^GA LMD/CS = Gestational age by the last menstruation date or by Capurro sommatic.

gestational age by Ballard.

The main diagnosis found among the 57 NBs admitted to the ICU are represented on Table 3. From these, 7 NBs failed the TOAEs in the first stage. This result bears statistical significance when compared to the NBs who did not stay in the ICU.

**Table 3:** NBs distribution in the ICU regardless of stay duration in relation to the TOAE results in the first stage.

TOAE						
	Passed		Failed		Total	
ICU	N	%	N	%	n	%
Yes	50	8.0	7	1.1	57	9.1
No	420	67.2	148	23.7	568	90.9
Total	470	75.2	155	24.8	625	100.0

X^2^ = 5.27 p = 0.02168

The risk indicators for congenital hearing loss seen in the study were analyzed separately, according to Table 4, Table 5.

**Table 4:** Distribution of the high risk factors for hearing loss among the NBs studied in relation to the results obtained from the TOAE test.

		TOAE	
HIGH RISK FACTOR	N (%)	Passed	Failed
Intraventricular hemorrhage	9 (1.4)	8	1
Mother HIV positive	5 (0.8)	3	2
ICU ≥ 2 days	47 (7.5)	41	6
Weight < 1.500g	18 (2.9)	14	4
Apgar 1° (1–3)	15 (2.4)	13	2
Apgar 5° (1–5)	5 (0.8)	5	0
Syndromes	6 (1.0)	2	4
Family history	38 (6.1)	29	9
Craniofacial anomalies	6 (1.0)	2	4
Toxoplasmosis	4 (0.6)	4	0
Syphilis	4 (0.6)	3	1
Cytomegalovirus	2 (0.3)	1	1
Mechanical ventilation	3 (0.5)	2	1
Drugs (tobacco. alcohol. illegal)	47 (7.5)	35	12
Ototoxic medication in gestation	46 (7.4)	36	10
Ototoxic medication in the NB	43 (6.8)	34	9

**Table 5:** Results from the TOAE tests in relation to the number of risk factors observed.

		TOAE	
Number of Risk Factors	Number of NBs	Passed	Failed
0	438	320	118
1	118	97	21
2	29	22	7
3	12	10	2
4	16	13	3
5	3	2	1
6	6	5	1
7	1	1	0
8	1	0	1
9	1	0	1
Total	625	470	155

X^2^= 3,6 p = 0,0578

The association of risk factors together showed a trend towards statistical significance to present a TOAE failure (Table 5).

There was no association between the use of ototoxic medication and the rate of TOAE failures (p>0.05) (Table 4, Table 6).

**Table 6:** Distribution of the main ototoxic drugs used in the NBs admitted to the neonatal ICU.

Ototoxic medication	n	%
Aminoglycosides	38	6,1
Ampicillin	37	5,9
Vancomycin	4	0,6
Furosemide	3	0,5
Others	14	2,2
Total	96	15.4

## DISCUSSION

According to the references and the quality indicators defined by the JCIH in 2000, in order for a NAS program to be universal, within six months of implementation it must reach a minimum of 95% of infants assessed during post-partum admission, or before one month of life.^3^^,^^5^ The NAS implementation study in this university hospital tracked 81.7% of the 765 NBs eligible for the test. This rate of scope, although it is not ideal, is very similar to the reality found in other services at this same implementation stage. Kennedy et al., in 2005, in a cohort study assessed 25,609 NBs in the United Kingdom in three years, showed that only 83% of these NBs were screened.^6^ Chapchap and Segre, in the year 2000, assessed 4.196 NBs, recording 90.6% of the NBs tested.^7^ Therefore, the 95% reference was not reached in the first six months of implementation.

The screening was carried out in 595 NBs (95.2%) during the stay in joint quarters at the Neonatal Unit. 17.96% of the NBs returned for outpatient testing, 30 of the 167 NBs expected. The rate of “no shows” at the outpatient ward screening first stage was of 82%. A study carried out in Malaysia, by Mukari et al.^8^, involving 4,437 newborns in the period of April, 2003 through February, 2004, shows an unsatisfactory scope rate in the first test (84.64%), low rate of outpatient return (11.97%) and return for a new test for those who failed (56.97%). Of the 16 NBs with hearing loss identified in the study, only one had suffered an intervention. In order to identify the factors which led to the unsatisfactory results in the program, the authors interviewed 314 mothers who did not return with their children after we found them through an active search. The main reason reported was a communication failure.

For the narrow scope in the first maternity test, Mukari et al. relate to hospital discharge before 24 hours after birth to the possibility of return after discharge, and the test performance was no longer a priority during hospital stay; and the fact that the examiners worked from Monday to Friday, and the test was not performed in those children to be discharged during the weekend.

On the NAS first stage, in this service, 458 NBs (73.3%) passed the TOAE and CPR tests and 167 (26.7%) failed, as per described on Table 1. Results point to a TOAE rate of failure of 24.8% (155 RNs) (Table 1). Pádua et al., 2005, observed a test positiveness 9.5% on the 1,127 NBs examined in a 9 month NAS study.^9^

As we analyzed the failures reported by Wroblewska-Seniuk et al. in 2005, we observed a positive test in 16.28% of the total of 5,601 NBs studied. Of these, 61.5% failed unilaterally on the TOAE test.^10^ The unilateral failures observed by Pádua et al. were of 67.3%^9^, and such values are similar to the ones observed in the present study, representing 62.6% of the positive TOAEs. In 2003, Segre mentions that the rate of UNAS retesting can reach up to 10%.^11^

It is a fact that the high rate of TOAE false-positives represent a disadvantage of this type of test. This NAS had 16.9% of false-positive emissions on the first stage of the assessment. Marone et al., 2005, reported 15.5% of false positive tests.^12^

The short hospital stay period after birth can raise the false-positive rate. The presence of amniotic liquid and caseous vernix in the auditory canal may impair sound transmission and cause failure in the emissions test.^10^ It is suggested that a serial repetition of the altered tests, still during hospital stay can reduce such problem. The test repetition would require a longer hospital stay for the NB.^10^^,^^13^^,^^14^ In the hospital of our study, children are discharged between 36 and 48 hours of life. Korres et al., in a recent publication from 2006, observed a failure rate in emissions in only 3.1% of the NBs in the first three years of the program. However, the children were kept in the hospital in the first 4 to 5 days of life.^13^

All the children with the first TOAE positive test and or without CPR, were referred for retesting in the lab. And, following the guidelines suggested by the JCIH, the NBs with some high risk indication concerning hearing loss, because of the risk of progressive loss or a loss of late onset, were referred for follow up every six months for three years.^3^^,^^12^ Of this group, 8 children returned for outpatient follow up.

For the second stage with 122 children, 114 were among the 167 NBs who failed the first stage, representing 68.2% of program compliance, that is, the children who failed the first stage and returned for retesting. This value is similar to the 73.1% described in the Durante et al study and more expressive than the 41.8% compliance observed by Korres et al. at the end of the fifth year of universal screening with TOAE only (Chart 2).^2^^,^^13^ Thus, a follow up above the 70% reference indicated by the JCIH in the beginning of the NAS implementation was near reach.^3^

On the second screening stage, 6.6% of the children failed the TOAE test. In the studies reported above, the authors observed higher failure rates in the retest with TOAE, from 23.81 to 39.91%.^10^^,^^2^^,^^13^

The behavioral assessment is considered an important part of the investigation as it provides information of how auditory resources are used by the child and for assessing central auditory pathways. The presence of behavioral information suggests integrity of the hearing pathways and no severe hearing loss.^12^

In the present investigation, CPR was absent in 6.1% of the time in the first screening stage and in 6.5% on the second. In the study carried out by Pádua et al. they found an index of 1.8% of CPR alterations at the UNAS.^9^ Analyzing only the CPR results on the second stage of the program under investigation, we noticed that four children in the study failed only the reflex behavioral evaluation, representing 0.6% of the study subjects.

12 children were referred for diagnostic investigation follow up, representing 1.9% of the NBs tested in the Neonatal Auditory Screening Program in six months and 1.6% of the live births eligible for hearing screening (765). The NAS quality indicator proposed by the JCIH indicates that at the end of the first year of the program, less than 4% of the NBs screened should be referred as a goal to avoid high false-positive rates.^3^

On the 12 children referred for the third stage of the follow up, we see the presence of at least one risk factor in 11 of them (Chart 1).

187 newborns with risk factors for hearing loss were identified, representing 29.92% of the sample. Of these, 37 newborns failed TOAEs (Table 5). The rate of emissions with failure, when associated with risk factors showed a tendency to having statistical significance. Some author report the presence of risk factors in 6.910, 12.52and 14.7%9of the NBs eligible to universal screening.

Nóbrega et al. described congenital rubella as one of the main etiologies in children, even showing a high percentage of loss for unknown cause.^15^ Congenital infections are described as being responsible for a large number of hearing losses in Brazil. Among them, we have infection by cytomegalovirus, the most common infection of the TORCH complex.^14^ Marone et al. found perinatal infections in 25.8% of the NBs investigated.^12^

Maternal infection by HIV can also be considered a risk factor because of vertical transmission.^12^ In our study, we found maternal infection in 0.8% of the cases. Chandrasekhar et al., in the year 2000 and Rezende et al., in 2004, point to possible direct effects of the virus in the inner ear, yet to be clarified among the causes of hearing loss in HIV-positive patients because of its neurotropic behavior and involvement of the cochleovestibular nerve. Another mechanism would be ototoxicity caused by antiretroviral drugs, zidovudine among them, used in the prophylactic treatment of NB from HIV-infected mothers.^16^, ^17^, ^18^, ^19^

One important factor observed in the sample is the presence of hearing loss in relatives of the first to the third grades (6.1%). Hereditary can represent up to 50% of hearing loss in childhood.^20^ Nóbrega et al. relate to genetic causes around 14% of the causes for hearing deficit in children and teenagers.^15^ It is believed that the high prevalence of mutations in the GJB2 gene justifies the inclusion of genetic screening to complement the current investigation approaches.^1^

It is estimated that the prevalence of hearing deficit in children with past history of ICU stay increase in 6 fold, because of the higher risks of comorbidities and complications.^15^ The myelinization of the auditory nerve fibers start on the 24th week of gestation, on the 26th week the Organ of Corti is already morphologically similar to that of the adult and the cochlear auditory evoked potentials are present as of the 24th week.^12^ For these reasons, we took into account the use of ototoxic medications during gestation, assuming a possible ototoxic effect for the fetus. The number of pregnant women with a medical chart who used ototoxic medication was of 46 (7.4%), and 21.7% failed the emissions (Table 4). There was no relationship between the use of ototoxic medication during gestation and the increase in the number of TOAE failure in the first stage (p>0.05).

Of the 12 children referred for a third stage investigation, five underwent brainstem audiometry test. Results were altered in two children, representing 0.32% of the study population (Chart 2). Hall et al., 2004, studying the combination of TOAE with BAEP, describing a 2% rate of positive screening.^21^, ^22^, ^23^

The goal established by the JCIH is reaching diagnosis before 3 months and perform intervention by 6 months of age. In the study program, hearing loss diagnosis of the two children was carried out between 7 and 10 months of age. They are being evaluated by the multidisciplinary auditory development follow up program for proper treatment. The children without BAEP test are being looked for, for testing.

After the screening results in 81.7% of the NBs eligible, we tried to identify the factors that could justify them. Checking all the births at the maternity from the Obstetric Center Records and crossing such data with those from NBs who had been submitted to screening, we noticed that of the births which happened on Thursdays and Fridays of NBs who did not undergo TOAEs before hospital discharge, 57.6% were born in these two days of the week. Of these, 20.7% came from other towns.

Korres et al. (2006) suggest the factors that can make an auditory screening program a success. During the first three years of the program implementation in Athens, the authors identified the probable causes of narrow scope. In the two following years, they continued to enhance the program, and currently, the program reached the metrics proposed by the JCIH. The first testing before discharge went from 58.9 to 96.3% and the return for tests in the outpatient ward increased from 27.8 to 41.8%. The authors relate the good results especially to the fact that NBs are being discharged between four and five days of life. The first test is carried out before 24 hours, and when it fails, it is repeated many times until the day of discharge. The program also includes tests during the 7 days of the week, training of all the professionals involved with the care of pregnant women, from prenatal care to the delivery, to the care given to the newborn all the way to their discharge and follow up. NBs who failed had their testing repeated many times until the time they went home is stressed by the authors as the main factor responsible for the program to have reached only 2.1% of fails in the first OAE. These NBs who could effectively have hearing loss and the need to follow the program were more easily followed in the continuation of the investigation and intervention. Another thing that was stressed was to alert the parents regarding the probable risk of hearing deficit in their children, since the test was carried out many times, engaging them even more emphatically regarding follow up after going home.^13^^,^^14^

Korres et al.^13^^,^^14^ and Mukari et al.^8^ reported good results after implementing some proposals to improve hearing screening programs.

During the study, the NAS in this university hospital completed one year in the beginning of the implementation. Some improvements started after focusing more intensively on the attempt to guarantee the performance of these tests before maternity discharge. Other improvements are still to be implemented in order to increase the rates of compliance and reduce the rates of false-positive.

The joint quarters team and the Neonatal Unit is more prepared to identify the NBs who have not yet been tested. The members of the medical team are alert as they discharge these children. As far as weekends and holidays are concerned, there is the need to create mechanisms for NBs with discharge scheduled for these days, either be tested before, or that the tests be performed even during these days by appointed professionals.

At the time of hospital discharge, we suggest to identify the charts of NBs who suffered the test and it was normal and the ones who failed, and who should return. The ones who were not registered, therefore were not submitted to the first TOAE, and are being discharged, may be identified later on, for active search in case they miss their return scheduled to the outpatient ward.

In order to better educate parents at the time of discharge, we suggest the creation of an educational card. In such a card, there must be the identification, data and time of hospital discharge; date, time and place of return; and also the telephone number of the program for the possibility of a new scheduling of the return visit when necessary.

The disclosure to the community by means of communication, the alert about the need to undergo hearing deficit screening as early as possible and the involvement of all health care professionals from a primary level, during prenatal care and during pediatric care, are strategies to educate parents regarding the importance of hearing screening. Parents may become inspectors of the program's effectiveness, as it happens today with the foot test.

Because of its repercussion on citizenship, hearing loss represents a public health problem. Health care authorities have the responsibility of stimulating the implementation of programs in the many cities, creating points of reference for easier access by the population. And after implementing them, means must be created to assure their real effectiveness.

The data from this study are related to the first six months of the program. There is the need to update the data in the program's sequence and check for the effective improvement on test rates in its many stages. The implementation of a neonatal hearing screening in a university hospital, which serves only the public health care system, bears innumerous difficulties. The involvement of all the members of the multidisciplinary team is paramount.

## CONCLUSIONS

The first 6 months of implementation of the Neonatal Hearing Screening Program in the University Hospital have an engaging rate of 81.7% of the eligible newborns. The compliance rate, in other words, return of newborns with TOAE failure was of 68.2%. Of the NBs tested, 1.9% required investigation by the BAEP and 0.32% had hearing loss.

Some important things were noticed at the time of implementation: team dedication and commitment, disclosure and family education, and their interest in such a program.

In order to improve the NAS program scope we propose: that the tests be repeated before hospital discharge, tests be carried out during the weekends and holidays, a greater integration of the health care team, distribute explanatory cards to the parents during hospital stay, better information on the press and greater governmental action in order to divulge NAS.

The program is still in its implementation phase; the difficulties found and others that will come must be constantly analyzed and the solutions put into practice in order to make the screening program truly universal.
